# Molecular Characteristics of Novel Phage vB_ShiP-A7 Infecting Multidrug-Resistant *Shigella flexneri* and *Escherichia coli*, and Its Bactericidal Effect *in vitro* and *in vivo*

**DOI:** 10.3389/fmicb.2021.698962

**Published:** 2021-08-26

**Authors:** Jing Xu, Ruiyang Zhang, Xinyan Yu, Xuesen Zhang, Genyan Liu, Xiaoqiu Liu

**Affiliations:** ^1^Key Laboratory of Pathogen Biology of Jiangsu Province, Nanjing Medical University, Nanjing, China; ^2^Department of Microbiology, Nanjing Medical University, Nanjing, China; ^3^State Key Laboratory of Reproductive Medicine, Nanjing Medical University, Nanjing, China; ^4^Department of Laboratory Medicine, The First Affiliated Hospital With Nanjing Medical University, Nanjing, China; ^5^National Key Clinical Department of Laboratory Medicine, The First Affiliated Hospital With Nanjing Medical University, Nanjing, China

**Keywords:** bacteriophage vB_ShiP-A7, genome sequence, multidrug resistant, mass spectrometry, comparative genome, bactericidal ability

## Abstract

In recent years, increasing evidence has shown that bacteriophages (phages) can inhibit infection caused by multidrug-resistant (MDR) bacteria. Here, we isolated a new phage, named vB_ShiP-A7, using MDR *Shigella flexneri* as the host. vB_ShiP-A7 is a novel member of *Podoviridae*, with a latency period of approximately 35 min and a burst size of approximately 100 phage particles/cell. The adsorption rate constant of phage vB_ShiP-A7 to its host *S. flexneri* was 1.405 × 10^–8^ mL/min. The vB_ShiP-A7 genome is a linear double-stranded DNA composed of 40,058 bp with 177 bp terminal repeats, encoding 43 putative open reading frames. Comparative genomic analysis demonstrated that the genome sequence of vB_ShiP-A7 is closely related to 15 different phages, which can infect different strains. Mass spectrometry analysis revealed that 12 known proteins and 6 hypothetical proteins exist in the particles of phage vB_ShiP-A7. Our results confirmed that the genome of vB_ShiP-A7 is free of lysogen-related genes, bacterial virulence genes, and antibiotic resistance genes. vB_ShiP-A7 can significantly disrupt the growth of some MDR clinical strains of *S. flexneri* and *Escherichia coli* in liquid culture and biofilms *in vitro*. In addition, vB_ShiP-A7 can reduce the load of *S. flexneri* by approximately 3–10 folds in an infection model of mice. Therefore, vB_ShiP-A7 is a stable novel phage with the potential to treat infections caused by MDR strains of *S. flexneri* and *E. coli.*

## Introduction

A growing number of multidrug-resistant (MDR) bacteria have been reported, and the emergence of MDR bacteria leads to serious systemic and biofilm-associated infections (Shiferaw et al., 2012; Ahmed and Shimamoto, 2015; Klontz and Singh, 2015). For example, infections caused by MDR *Enterobacteriaceae*, especially β-lactam-resistant *Enterobacteriaceae*, are difficult to treat (De Angelis et al., 2020). *Shigella* spp. and *Escherichia coli* are major members of the family *Enterobacteriaceae*, which comprises important enteric pathogens (The et al., 2016; Kotloff et al., 2018). *Shigella* spp. and *E. coli* can cause human infection through food contamination; in particular, a small number of cells of *Shigella* can cause diarrhea in humans (The et al., 2016). Globally, the morbidity and mortality rates of diarrhea caused by *Shigella* are high. In the era of antibiotic crisis, bacteriophages have been demonstrated to be effective alternative biocontrol agents for these members of *Enterobacteriaceae* (Kakasis and Panitsa, 2019).

Phages were first used to treat diarrhea caused by *Shigella* in the 1920s (D‘Herelle, 1923). However, after antibiotics were discovered and widely used, only Eastern European countries insisted on phage treatment for infections (Nikolich and Filippov, 2020). In the 21st century, phages against MDR *Shigella* have been widely investigated (Tang et al., 2019; Kortright et al., 2019). Bernasconi et al. proved that three commercially available bacteriophage cocktails could suppress *Shigella* infections in the human intestine (Bernasconi et al., 2018). In a mouse model, Mai et al. prepared a new reagent composed of a phage cocktail and an antibiotic (ampicillin), named ShigActive^TM^, to lyse *Shigella* (Mai et al., 2015). *Shigella* phages have also been successfully applied to food safety (Zhang et al., 2013). Soffer et al. characterized five lytic bacteriophages and combined them as a cocktail, ShigaShield^TM^, to inhibit the growth of *S. sonnei* in food—the process of FDA and USDA assessment of the GRAS status (GRN672) (Soffer et al., 2017). Several phages have been reported to have the ability to infect both *E. coli* and *Shigella* (Chang and Kim, 2011; Lee et al., 2016; Sváb et al., 2019). Phages have been widely studied in the prevention and treatment of infections caused by *Shigella* spp. and *E. coli* in humans, suggesting that phages are promising for the treatment of infections caused by these *Enterobacteriaceae* members.

There are many limitations in phage therapy, such as phage pharmacology and infection kinetics, a narrow host range of phages, and phage resistance to bacteria. It has been shown that phage cocktails can overcome the limitations of the narrow host range of phages and phage resistance to bacteria (Matsuzaki et al., 2014; Kakasis and Panitsa, 2019). Cocktails of well-known lytic phages might open new perspectives in controlling infections caused by MDR bacteria. Therefore, isolating and characterizing more phages will allow us to obtain enough stock phages for the preparation of personalized phage cocktails against different clinical MDR bacterial strains. In addition, a comprehensive study of each novel phage is required to exclude a phage encoding toxic proteins or lysogen-related proteins in phage cocktails. It is also necessary to evaluate the effectiveness and safety of these new phages against MDR bacteria both *in vitro* and *in vivo*.

Phages have been isolated from different environmental sources and fecal samples of humans and animals (Kim et al., 2010; Sun et al., 2013; Jun et al., 2013; Schofield et al., 2015; Doore et al., 2018). In this study, we isolated a novel lytic phage named vB_ShiP-A7, using a clinically isolated MDR *S. flexneri* strain as a host, from wastewater in Nanjing, China. Phage vB_ShiP-A7 can also infect several clinically isolated MDR *E. coli* strains. The phage has a strong ability to lyse bacteria in liquid culture media and biofilms. Furthermore, phage vB_ShiP-A7 can significantly reduce the number of *S. flexneri* in mice. Thus, this phage may be used to monitor, diagnose, and control infections caused by MDR *S. flexneri* and *E. coli*.

## Materials and Methods

### Bacterial Strains

All of the bacterial strains used in this study were grown in Luria-Bertani (LB) medium at 37∘C (Table 1). *Escherichia coli* wild-type strain MG1655 was a stock of our lab, where as *S. flexneri* A7, *S. sonnei* A5, and 29 clinical strains of *E. coli* were isolated and cultured from different specimens of patients in the First Affiliated Hospital of Nanjing Medical University, Nanjing, China. Twenty-four clinical strains of *Shigella* spp. were tested at the Jiangsu Provincial Center for Disease Control and Prevention. *Shigella flexneri* A7 was deposited in the China Center for Type Culture Collection (CCTCC Number is PB 2020012) in Wuhan, China.

**TABLE 1:** Host range spectrum of the bacteriophage vB_ShiP-A7.

**Strains**	**Source**	**Subtype**	**Resistance**	**Lysis or not**	**Efficiency of Plating (EOP)**
*E. coli* K-12 MG1655	ATCC 700926			N	N
*Shigella flexneri* A7			Ampicillin sulbactam, Aminoglycoside streptomycin, Spectinomycin	Clear plaque	100%
*Shigella sonnei* A5			Spectinomycin, Gentamycin, Streptomycin, Aminoglycoside, Ampicillin sulbactam	N	N
*Shigella sonnei* S20001			Amikacin, Azithromycin, Streptomycin	N	N
*Shigella flexneri* S20003		2b	Amikacin, Ampicillin, Ampicillin-Sulbactam, Azithromycin, Cefoxitin, Chloramphenicol, Ciprofloxacin, Nalidixic Acid, Streptomycin, Tetracycline	N	N
*Shigella sonnei* S20004			Amikacin, Ampicillin, Azithromycin, Cefotaxime, Cefoxitin, Nalidixic Acid, Streptomycin, Tetracycline, Trimethoprim-Sulfamethoxazole	N	N
*Shigella flexneri* S20005		2a	Amikacin, Ampicillin, Ampicillin-Sulbactam, Azithromycin, Aztreonam, Cefotaxime, Cefoxitin, Chloramphenicol, Ciprofloxacin, Nalidixic Acid, Streptomycin, Tetracycline, Trimethoprim-Sulfamethoxazole	N	N
*Shigella flexneri* S20006		2a	Amikacin, Ampicillin, Ampicillin-Sulbactam, Azithromycin, Aztreonam, Cefotaxime, Cefoxitin, Ceftazidime, Chloramphenicol, Ciprofloxacin, Nalidixic Acid, Streptomycin, Tetracycline	N	N
*Shigella flexneri* S20008		2a	Amikacin, Ampicillin, Ampicillin-Sulbactam, Azithromycin, Cefoxitin, Chloramphenicol, Ciprofloxacin, Nalidixic Acid, Streptomycin, Tetracycline	N	N
*Shigella flexneri* S20009		1a	Amikacin, Ampicillin, Ampicillin-Sulbactam, Azithromycin, Cefoxitin, Chloramphenicol, Ciprofloxacin, Nalidixic Acid, Streptomycin, Tetracycline	Clear plaque	26.5%
*Shigella sonnei* S20010			Amikacin, Azithromycin, Cefoxitin, Streptomycin, Tetracycline,	N	N
*Shigella flexneri* S20012		1a	Amikacin, Ampicillin, Ampicillin-Sulbactam, Azithromycin, Cefotaxime, Cefoxitin, Chloramphenicol, Ciprofloxacin, Nalidixic Acid, Streptomycin, Tetracycline, Trimethoprim-Sulfamethoxazole	Clear plaque	24.6%
*Shigella flexneri* S20013		2a		N	N
*Shigella flexneri* S20014		2b	Amikacin, Azithromycin, Cefoxitin, Streptomycin,	N	N
*Shigella flexneri* S20015		2b	Amikacin, Azithromycin, Cefoxitin, Streptomycin,	N	N
*Shigella flexneri* S20016		2b	Amikacin, Azithromycin, Meropenem, Streptomycin,	N	N
*Shigella flexneri* S20017		2b	Amikacin, Azithromycin, Cefoxitin, Streptomycin,	N	N
*Shigella flexneri* S20018		2b	Azithromycin, Cefoxitin, Streptomycin,	N	N
*Shigella sonnei* S20020			Amikacin, Ampicillin, Azithromycin, Cefotaxime, Cefoxitin, Nalidixic Acid, Streptomycin, Tetracycline, Trimethoprim-Sulfamethoxazole	N	N
*Shigella flexneri* S20022		1a	Amikacin, Ampicillin, Ampicillin-Sulbactam, Azithromycin, Cefoxitin, Chloramphenicol, Ciprofloxacin, Nalidixic Acid, Streptomycin, Tetracycline	Clear plaque	38.7%
*Shigella flexneri* S20023		1a	Amikacin, Ampicillin, Ampicillin-Sulbactam, Azithromycin, Aztreonam, Cefoxitin, Ceftazidime, Ciprofloxacin, Nalidixic Acid, Streptomycin, Tetracycline, Trimethoprim-Sulfamethoxazole	Clear plaque	45%
*Shigella flexneri* S20024		2a	Amikacin, Ampicillin, Ampicillin-Sulbactam, Azithromycin, Cefoxitin, Chloramphenicol, Ciprofloxacin, Nalidixic Acid, Streptomycin, Tetracycline	N	N
*Shigella flexneri* S20025		2b	Amikacin,Azithromycin, Cefoxitin, Streptomycin,	N	N
*Shigella sonnei* S20026			Amikacin, Ampicillin, Azithromycin, Cefotaxime, Cefoxitin, Nalidixic Acid, Streptomycin, Trimethoprim-Sulfamethoxazole	N	N
*Shigella flexneri* S20027		1a	Amikacin,Azithromycin,Cefoxitin,Streptomycin	Clear plaque	38.9%
*Shigella flexneri* S20028	,	2a	Amikacin, Ampicillin, Ampicillin-Sulbactam, Azithromycin, Cefoxitin, Chloramphenicol, Ciprofloxacin, Nalidixic Acid, Streptomycin	N	N
*Shigella flexneri* S20029		1a	Amikacin,Azithromycin, Cefoxitin, Streptomycin	Clear plaque	41.1%
*E. coli* 393 D3	Urine	ESBL	Levofloxacin, Cefazolin, Cefepime, Cefotaxime, Ceftazidime	Clear plaque	0.03%
*E. coli* 395B5	Urine	ESBL	Gentamycin, Ampicillin sulbactam, Aztreonam, Cefepime, Cefazolin, Levofloxacin, Cefotaxime, Sulfamethoxazole and Trimethoprim	Clear plaque	4.1%
*E. coli* 397 D3	Urine	ESBL	Gentamycin_‵_Ampicillin sulbactam_‵_Levofloxacin_‵_Cefotaxime, Cefepime, Aztreonam, Cefoxitin, Cefazolin, Sulfamethoxazole and Trimethoprim	Turbid plaque	N
*E. coli* 389 A6	Urine	ESBL	Sulfamethoxazole and Trimethoprim, Cefazolin, Aztreonam, Ceftazidime	N	N
*E. coli* 389G7	Urine	ESBL	Ampicillin sulbactam, Aztreonam, Cefazolin, Amikacin, Levofloxacin, Cefotaxime	N	N
*E. coli* 393B7	Urine	ESBL	Levofloxacin, Gentamycin, Cefazolin, Ampicillin sulbactam, Ceftazidime, Amoxicillin and Clavulanate, Amikacin, Aztreonam, Sulfamethoxazole and Trimethoprim, Minocycline, Cefotaxime, Cefepime, Cefoxitin	N	N
*E. coli* 393C8	Urine	ESBL	Sulfamethoxazole and Trimethoprim, Cefepime, Cefazolin, Cefotaxime, Levofloxacin	N	N
*E. coli* 395G6	Urine	ESBL	Gentamycin, Aztreonam, Cefazolin, Cefepime, Ceftazidime, Cefotaxime	N	N
*E. coli* 396J1	Urine	ESBL	Levofloxacin, Cefazolin, Imipenem, Cefepime, Cefotaxime	N	N
*E. coli* 394H7	Urine	ESBL	Cefotaxime, Levofloxacin, Ceftazidime, Aztreonam, Cefepime	N	N
*E. coli* 396J5	Urine	ESBL	Levofloxacin, Aztreonam, Imipenem Cefepime, Cefazolin, Cefotaxime	N	N
*E. coli* 389 G7	Urine	ESBL	Cefazolin, Ampicillin sulbactam, Levofloxacin, Aztreonam, Amikacin, Cefotaxime	N	N
*E. coli* 394F7	Urine	ESBL	Cefazolin, Aztreonam, Cefoxitin, Ceftazidime, Cefotaxime, Amoxicillin, and Clavulanate	N	N
*E. coli* 389D9	Urine	Non-ESBL	Sulfamethoxazole and Trimethoprim	N	N
*E. coli* 389G6	Urine	Non-ESBL	Levofloxacin	N	N
*E. coli* 389G4	Urine	Non-ESBL	Gentamycin, Cefazolin, Ampicillin sulbactam, Cefotaxime, Amikacin, Levofloxacin, Ceftazidime, cefoxitin	N	N
*E. coli* 390B6	Urine	Non-ESBL	Sulfamethoxazole and Trimethoprim	N	N
*E. coli* 390J2	Urine	Non-ESBL	Minocycline, Levofloxacin, Sulfamethoxazole, and Trimethoprim	N	N
*E. coli* 389E6	Urine	Non-ESBL	Levofloxacin	N	N
*E. coli* 390G7	Urine	Non-ESBL	Sulfamethoxazole and Trimethoprim, Gentamycin, Cefazolin, Ampicillin sulbactam, Levofloxacin	N	N
*E. coli* 390H2	Urine	Non-ESBL	Ampicillin sulbactam, Levofloxacin, Sulfamethoxazole and Trimethoprim, Gentamycin, Cefazolin	N	N
*E. coli* 391D3	Urine	Non-ESBL	Gentamycin, Cefazolin, Sulfamethoxazole and Trimethoprim	N	N
*E. coli* 389J4	Sputum	ESBL	Cefepime, Ceftazidime, Cefazolin, Aztreonam	N	N
*E. coli* 395J2	Sputum	ESBL	Gentamycin, Ampicillin, Minocycline, Levofloxacin	N	N
*E. coli* 397C8	Sputum	ESBL	Ceftazidime, Aztreonam, Cefepime, Ceftazidime	N	N
*E. coli* 396F3	Sputum	ESBL	Ampicillin, Gentamycin, Minocycline, Levofloxacin, Amikacin	N	N
*E. coli* 390A7	Sputum	Non-ESBL	Sulfamethoxazole and Trimethoprim	N	N
*E. coli* 391G4	Blood	ESBL	Cefepime, Cefazolin, Aztreonam, Ceftazidime	N	N
*E. coli* 393C1	Ascites	ESBL	Cefotaxime, Cefazolin, Cefepime, Ampicillin sulbactam, Aztreonam, Gentamycin, Ceftazidime, Sulfamethoxazole, and Trimethoprim	N	N

*Negative results are indicated by “N.”*

### Isolation and Propagation of Bacteriophages

vB_ShiP-A7 was screened from wastewater in Nanjing (China) using an MDR strain *S. flexneri* A7 as a host. First, a wastewater sample was filtered through a 0.45-μm filter (Millipore, United States). Thereafter, the filtered liquid was added to the early-log-phase culture of *S. flexneri* A7 and cultured at 37∘C for 4 h to enrich the phages. The cell culture was spun down to remove bacterial cells. Next, 10 μL of the supernatant, 100 μL of *S. flexneri* A7, and 3 mL of 0.6% (w/v) LB agar were mixed well, and the mixture was poured on the surface of an LB plate. After culturing at 37∘C for approximately 12 h, plaques formed on the plate. A single clear plaque was selected to start a new round of screening. After several rounds of screening, the plaques appeared homogeneous on a double-layer agar plate. A single clear plaque was selected and inoculated in a liquid culture of the host strain. The supernatant of the culture with the phage was filtered through a 0.22 μm filter and used to start a new round of screening. We again obtained homogeneous plaques on a double-layer agar plate. The preliminary purified phage, named vB_ShiP-A7, from a single plaque was obtained and stored at 4∘C.

### Purification of Phage vB_ShiP-A7

Phage vB_ShiP-A7 was purified following the protocol of Yu et al. (2017). Briefly, phage vB_ShiP-A7 was added to the early-log-phase liquid culture of *S. flexneri* A7. After incubation at 37∘C for 2 h, the culture medium was spun down at 14,000 × *g* for 10 min at 4∘C. The supernatant was collected and passed through a 0.45 μm filter. The filtrate was concentrated using ultrahigh-speed centrifugation. The supernatant was removed, and the pellet was resuspended in SM buffer (10 mM Tris-HCl, pH 7.5; 100 mM NaCl; and 10 mM MgSO_4_). The suspension was further separated using cesium chloride gradient ultrahigh-speed centrifugation. We collected approximately 1 mL of phage zone and diluted it 10 times in SM buffer. The sample was then centrifuged at 200,000× *g* for 3 h to remove cesium chloride. The pellets were resolved in SM buffer as the purified phage particles of vB_ShiP-A7.

### Electron Microscopy

A drop of purified phage vB_ShiP-A7 particles (2 × 10^11^ PFU/mL) was dripped onto a copper grid. The phages on the copper grid were negatively stained using 2% (w/v) phosphotungstic acid. The morphology of phage vB_ShiP-A7was observed using an FEI Tecnai G2 Spirit Bio TWIN transmission electron microscope at 80 kV.

### Analysis of the Phage Host Range

The infection ability of phage vB_ShiP-A7 on different strains was determined using standard spot tests (Kutter, 2009). Briefly, 100 μL of log-phase bacterial culture of each strain was mixed with 3 mL of melted soft agar (0.6% agar), which was poured over an LB plate. After preparing different concentrations of phagevB_ShiP-A7 suspensions (10^10^–10^2^ PFU/mL), 5 μL of each concentration of the phage suspension was dropped onto the surface of solidified plates containing different test strains. After overnight culture at 37 C, the inhibition of bacterial growth by different concentrations of vB_ShiP-A7 on each plate reflected the sensitivity of the strain to vB_ShiP-A7. All experiments were conducted in accordance with the ethical guidelines of Nanjing Medical University, the First Affiliated Hospital of Nanjing Medical University, and Jiangsu Provincial Center for Disease Control and Prevention (Nanjing, China), and informed consent was obtained from all patients.

### Efficiency of Plating (EOP)

This method has been previously described by Kutter (2009). The positive clinical strains screened via standard spot tests were plated on double-layer agar plates with the phage. Plaques on each plate were counted, and the relative EOP was given as the ratio between the number of plaques on each clinical strain and the number of plaques on the host strain.

### Temperature Stability

The thermal stability of phage vB_ShiP-A7 was determined at different temperatures. Five test tubes containing 10^9^ phage particles were immersed in different temperature water baths for approximately 1 h (4, 25, 37, 45, and 50∘C). Thereafter, the phage titers of all the samples were determined using the double-layer agar method (Yu et al., 2017). Three independent repeated experiments were performed. The average value was used to generate the figure, and the standard error of the mean (SEM) was marked.

### Phage Adsorption Rate

The phage adsorption rate was determined using the protocol described by Zurabov and Zhilenkov (2021). Cells from an overnight culture of *S. flexneri* A7 were suspended in an LB broth to obtain a cell density of 10^8^ CFU/mL. The phage suspension was added to 5 mL of the bacterial suspension to a final concentration of 10^7^ PFU/mL. Thereafter, the mixture of phage and bacteria was diluted in 45 mL LB broth and incubated at 37∘C and 100 rpm for 5 min. The supernatant was filtered through a 0.22 μm filter, and the concentration of the free phages was determined using the plaque assay described above. The reduction in phage titer was equal to the number of phages adsorbed to the cells. The phage titer did not change in the control phage filtrate. We calculated the adsorption constant of the phage using the formula shown below (Zurabov and Zhilenkov, 2021), where k is the adsorption rate constant, P is the concentration of free phage per mL, P0 is the initial concentration of phage, B is the bacterial density, and t is the time in minutes over which adsorption occurs.

$$\text{k}=-\ln\,\left(\frac{\text{P}}{\mathrm{P0}}\right)/\mathrm{Bt}$$

### One-Step Growth Curve of Phage vB_ShiP-A7

A one-step growth curve of phage vB_ShiP-A7 was drawn following the protocol of Yang et al. (2015), with minor modifications. Phage vB_ShiP-A7 was added to the early-log-phase of the *S. flexneri* A7 culture (1 × 10^8^ CFU/mL) at a multiplicity of infection (MOI) of 0.1 and incubated at 37∘C for 5 min. The phages were then removed through centrifugation. Thereafter, the precipitate was put into 50 mL of LB medium and cultured at 37∘C for 5 min. We obtained 1 mL cell cultures at different time points and centrifuged them at 14,000 rpm for 1 min (Eppendorf Centrifuge 5424) to remove the host bacteria. Free bacteriophages in these supernatants were counted using the double-layer agar plate method. Three independent experiments were performed to obtain the one-step growth curve of vB_ShiP-A7, in which the latency period, burst period, and burst size of the vB_ShiP-A7 were determined. The latency period refers to the time from the adsorption of bacterial cells by the phage to the release of new phages from the bacterial cells. The burst period refers to the period from the beginning of phage release to the end of phage release, which is just after the latency period. The burst size of the phage is equal to the amount of phage at the end of lysis divided by the initial number of bacterial cells at the time of infection.

### Bacterial Challenge Assay

Overnight culture of *S. flexneri* A7 was added into the LB medium at a ratio of 1:100 and cultured for 2.5 h to the logarithmic phase. Phage vB_ShiP-A7 (MOI = 10, 1, or 0.1) was added to the log-phage cultures of *S. flexneri* A7. An equal volume of SM buffer was added to the negative control sample. We measured the optical density at 600 nm (OD600) of the bacterial culture medium every 15 min for a total of 300 min.

### Genome Isolation and Sequencing of Phage vB_ShiP-A7

The ultra-purified particles of vB_ShiP-A7 (approximately10^11^ phage particles) were digested by DNase I (New England Biolabs) and RNase A (Tiangen Biotech) at 37∘C for 2 h to remove the residual genomic DNA and RNA of the host bacteria. Thereafter, the sample was treated with proteinase K (Tiangen Biotech) at 55∘C for 15 min. This sample was further purified using a TIANamp Bacteria DNA Kit (Tiangen Biotech). The purified phage DNA concentration was measured using a spectrophotometer (Nanodrop Technologies, United States). The entire genome was sequenced on an Illumina platform (Illumina HiSeq 2500 sequencer, paired-end sequencing run, 2 × 150 bp). SOAPdenovov2.04 and GapCloser v1.12 were used to analyze the high-throughout sequencing results and assemble reads into a whole genome. The purified phage genome DNA was digested by the restriction endonuclease EcoRI or PstI at 37∘C for 6 h. The enzyme digestion products were analyzed using 1% agarose gel.

### Annotation and Comparison

Artemis software (Carver et al., 2005) and Glimmer 3 (Aggarwal and Ramaswamy, 2002) were used to predict the putative open reading frames (ORFs) in the genome of vB_ShiP-A7; each predicted protein length is more than 30 amino acids. Functional annotation of the genome of vB_ShiP-A7 was conducted using BLAST tools at NCBI^1^ against the non-redundant protein sequence database. tRNAscan-SE was used to find transfer RNAs (tRNAs) in the genome of vB_ShiP-A7(v1.23^2^). RNAmmer was used to find ribosomal RNAs (rRNAs) in the genome of vB_ShiP-A7 (v1.2^3^). Molecular masses and isoelectric points of all the predicted phage proteins were calculated using DNAman. Using the NCBI database, we found that the whole-genome sequence of several phages had high homology with phage vB_ShiP-A7 sequence (>40%). The EMBOSS needle tool was used to compare the similarity of protein amino acid sequences (European Molecular Biology Laboratory-European Bioinformatics Institute). EasyFig was used to compare the annotated proteins of vB_ShiP-A7 with those of related phages^4^ (Sullivan et al., 2011). The neighbor-joining algorithm in MEGA was used to analyze the phylogenetic relationships among the phages.

### Analysis of Phage Particle Proteins

The particles of vB_ShiP-A7 were mixed with loading dye and boiled in a 100°C water bath for 5 min. The boiled sample was separated using 12% sodium dodecyl sulfate-polyacrylamide gel electrophoresis (SDS-PAGE). The gel was stained with silver according to the protocol of Shevchenko et al. (1996). Liquid chromatography-electrospray ionization tandem mass spectrometry (LC-ESI MS/MS) was used to analyze the proteins in the particles of vB_ShiP-A7. The virions of vB_ShiP-A7 were digested with trypsin first, and the tryptic peptides were then analyzed using a Q Exactive mass spectrometer (Thermo Scientific, United States). The MASCOT engine was used to find the corresponding peptides (Matrix Science, London, United Kingdom; version 2.2) against all putative ORFs predicted in the genome of vB_ShiP-A7.

### Biofilm Biomass Quantification Using Crystal Violet Staining

Overnight bacterial cultures of *S. flexneri* A7 and *E. coli* 395B5were subcultured into LB medium separately until the early mid-logarithmic phase with approximately 10^8^ CFU/mL. The bacterial culture was diluted to 10^7^ CFU/mL using LB medium, and 200 μL of the diluted bacterial culture (10^7^ CFU/mL) was added to each well in a 96-well plate (Corning Corp., United States). After incubation at 37∘C for 24 h, 100 μL of the culture medium was removed from each well. At the same time, 100 μL of fresh LB medium with phage vB_ShiP-A7 (10^9^ PFU/mL) was added to the sample wells, and 100 μL of LB was added to the control wells. After culture for another 24 h, the culture medium of each well was gently removed. The wells were then washed twice using 1 × phosphate-buffered saline (PBS). Biofilms attached to the wells were stained with 0.5% (w/v) crystal violet (200 μL/well) for 20 min at 37∘C. To remove the excess crystal violet stain, the wells were washed 3 times with PBS. Pictures of the different wells were taken under a 20 × objective lens using a Zeiss (Axio Vert A1) microscope. Finally, the prestained biofilm was dissolved using 30% (v/v) acetic acid in water for biomass quantification at an absorbance of 560 nm using a plate reader (BioTek-Synergy HT).

### Inhibitory Effect of the Phage on *S. flexneri* in a Mouse Infection Model

Forty C57BL/6 mice (6 weeks old) were randomly divided into four groups: “control group,” “phage group,” “*Shigella* infection group,” and “*Shigella* infection plus phage treatment group,” with 10 mice in each group. Before infection with *S. flexneri* A7, the mice were fed drinking water containing broad-spectrum antibiotics (metronidazole 215 μg/L, colistin 0.85 U/mL, gentamicin 35 μg/L, and vancomycin 45 μg/L) for 3 days. On the fourth day, we started to feed these mice without antibiotics, and each mouse was weighed and recorded as the initial weight. On the fifth day, the day of mouse infection, *S. flexneri* A7 was grown to the exponential growth phase in an LB broth before being pelleted via centrifugation and washed 2 times in PBS. The bacteria were resuspended in the requisite volume of PBS to yield the desired in oculum (10^8^ CFU/mL). The mice in the “*Shigella* infection group” and “*Shigella* infection plus phage treatment group” were given 200μL bacterial suspension by gavage. The mice in the “control group” and “phage group” were given 200 μL of PBS by gavage. Five hours after bacterial infection, the purified phages were administered through drinking water to the mice in the “phage group” and “*Shigella* infection plus phage treatment group” (10^11^purified phage particles in SM buffer were added to 200 mL drinking water). The mice in the “control group” and *“Shigella* infection group” only received 200 mL drinking water with the same amount of SM buffer. After bacterial infection, the activity and defecation of these mice were observed. The feces of the mice were harvested, dissolved, and diluted. For the feces of each mouse, *S. flexneri* A7 was quantified via colony counts, and the phages was quantified using the plaques on the double-layer agar plates.

### Nucleotide Sequence Accession Number

The whole assembled genome sequence of vB_ShiP-A7 is deposited in the GenBank database under accession number MK685668.

## Results

### Morphology of Phage vB_ShiP-A7

**FIGURE 1:**
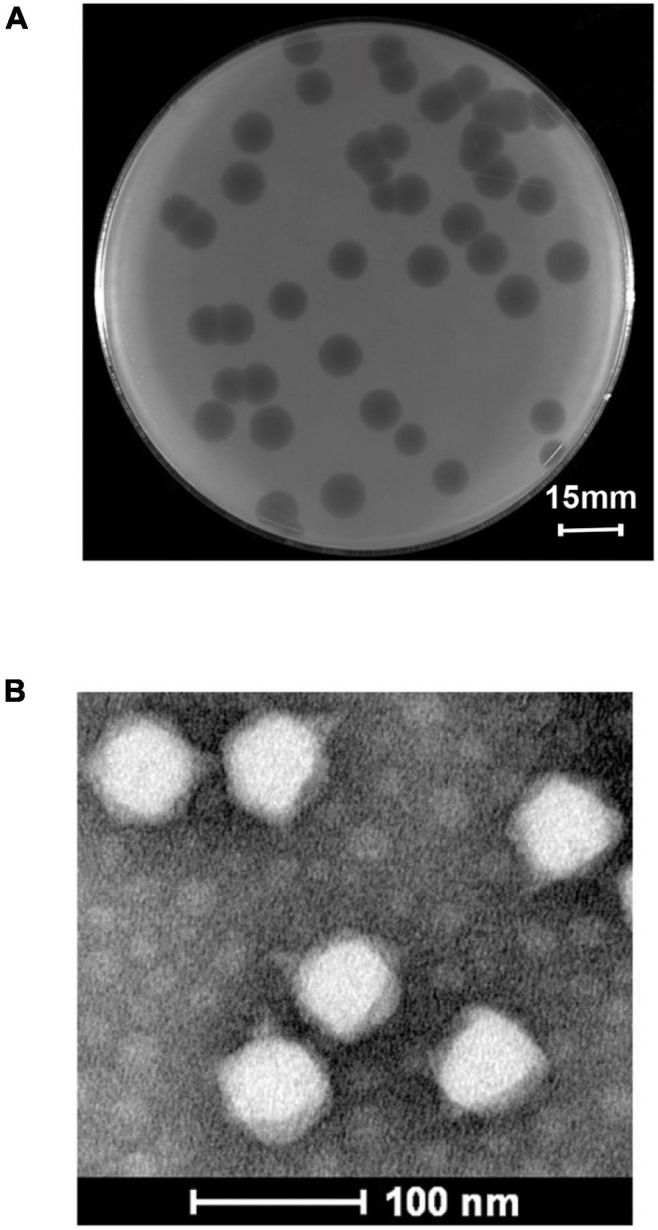
Isolation and morphological analysis of phage vB_ShiP-A7. **(A)** Plaques of phage vB_ShiP-A7 on the top agar plate. The bar indicates 15 mm. **(B)** The morphology of phage vB_ShiP-A7 under transmission electron microscopy. The bar indicates 100 nm.

Using *S. flexneri* A7 as the host strain, a novel phage vB_ShiP-A7 was isolated from wastewater in Nanjing, China. After overnight incubation with *S. flexneri* A7 at 37∘C, phage vB_ShiP-A7 formed large clear round plaques of approximately13 ± 0.60 mm in diameter on a double-layer agar plate (Figure 1A). We observed the morphology of this phage under an electron microscope. Phage vB_ShiP-A7 has an isometric head with a diameter of approximately 61.42 ± 1.23 nm and a noncontractile short tail of approximately 13 nm in length (Figure 1B). Phage vB_ShiP-A7 is a member of the family *Podoviridae*. This phage was named vB_ShiP-A7 following the phage nomenclature defined by Kropinski et al. (2009).

### Thermal Stability and Population Dynamics of Phage vB_ShiP-A7

**FIGURE 2:**
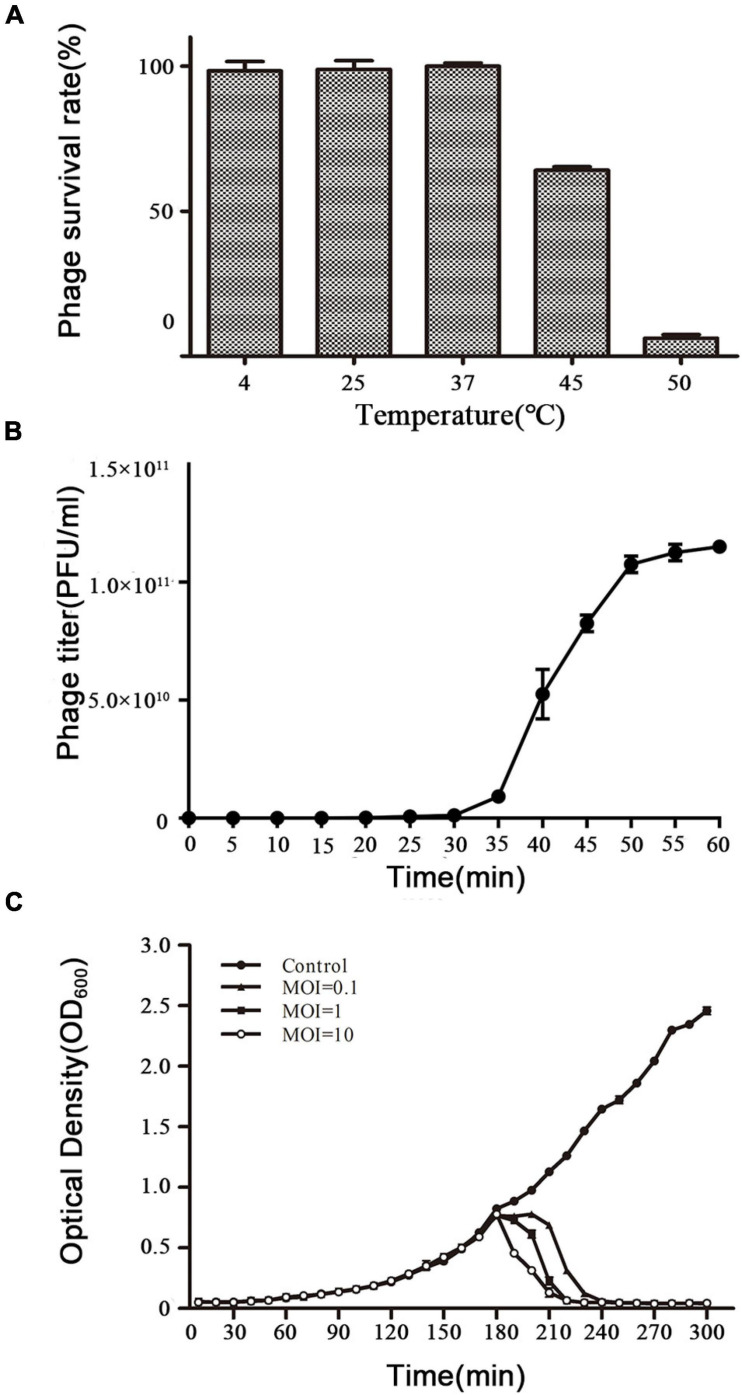
Biological characteristics of phage vB_ShiP-A7. **(A)** The survival rate of vB_ShiP-A7 after treatment at different temperatures for 1 h. **(B)** One-step growth curve of vB_ShiP-A7 on strain *Shiglla flexneri* A7 at 37∘C. **(C)** Bacterial challenge assay with phage vB_ShiP-A7 to *Shiglla flexneri* A7. vB_ShiP-A7 was added at MOIs of 0.1 (close triangles), 1 (close squares), and 10 (open circles) to different bacterial cultures after 2 h incubation (OD600 = 0.25). SM buffer instead of vB_ShiP-A7 was inoculated into a bacterial culture and used as the negative control (close circles). The light absorption value at OD600nm of each bacterial culture was recorded at 15 min intervals over a period of 300 min. In **(B,C)**, data from 3 independent experiments are combined and presented as the mean with the Standard Error of Mean (SEM).

The activity of vB_ShiP-A7 was measured after incubation at different temperatures (4∘C, 25∘C, 37∘C, 45∘C, and 50∘C) for 1 h (Figure 2A). The activity of phage vB_ShiP-A7 did not change from 4 to 37∘C. When the temperature increased up to 45∘C, the phage began to lose its activity rapidly (Figure 2A). These data suggest that vB_ShiP-A7 is stable over a relatively wide temperature range from 4 to 37∘C. Therefore, it can be preserved well at 4∘C in the laboratory as well as in the body of mammals at 37∘C. A one-step growth experiment was conducted to assess the population kinetics of phage vB_ShiP-A7 using *S. flexneri* A7 as a host (Figure 2B). Phage vB_ShiP-A7 was released 35 min after infection, with a burst size of approximately 100 phage particles/infected cell (Figure 2B). The adsorption rate constant at 5 min post-infection was determined to be 1.405 × 10^–8^ mL/min (Supplementary Table 1). Approximately 91.97% of the phage particles were adsorbed on the *S. flexneri* A7 cells in 5 min at an MOI = 0.1.

### Bacteriophage vB_ShiP-A7 Inhibits Planktonic Bacterial Growth

The efficacy of phage vB_ShiP-A7 on planktonic bacterial growth was assessed by inoculating a bacterial broth culture of *S. flexneri* A7 in the logarithmic period with phage vB_ShiP-A7 at different MOIs of 0.1, 1, and 10. Our results showed that the growth of the host strain *S. flexneri* A7 was completely disrupted within 90 min by phage vB_ShiP-A7 at different MOIs, and this growth inhibition lasted until 300 min after infection. The speed of bacterial lysis was accelerated by the increase in the phage titer (Figure 2C).

### Host Range and EOPs of Phage vB_ShiP-A7

The ability of phage vB_ShiP-A7 to infect different bacterial strains was estimated using standard spot tests (Kutter, 2009). Phage vB_ShiP-A7 can infect and form clear plaques on the MDR strain of *S. flexneri* A7 and on the 6 clinical MDR strains of *S. flexneri* serotype 1a but not the MDR strains of *S. Sonnei* (Table 1). In addition, vB_ShiP-A7 at low titers can form clear plaques on 2 of the MDR *E. coli* strains (393D3 and 395B5) and turbid plaques on 1 MDR *E. coli* strain (397D3); however, it cannot infect the wild-type *E. coli* strain MG1655 (Table 1). The EOPs of phage vB_ShiP-A7 on different clinical MDR strains are shown in Table 1. The results showed that the phage could specifically infect some serotypes of *Shigella* and also recognize some strains of *E. coli*, but it had a better lytic effect on strains of *Shigella flexneri* serotype 1a (Table 1). Thus, vB_ShiP-A7 may be used as a biocontrol agent to prevent or treat infections caused by MDR *S. flexneri* or *E. coli*.

### Basic Characteristics of the Genome of vB_ShiP-A7

**FIGURE 3:**
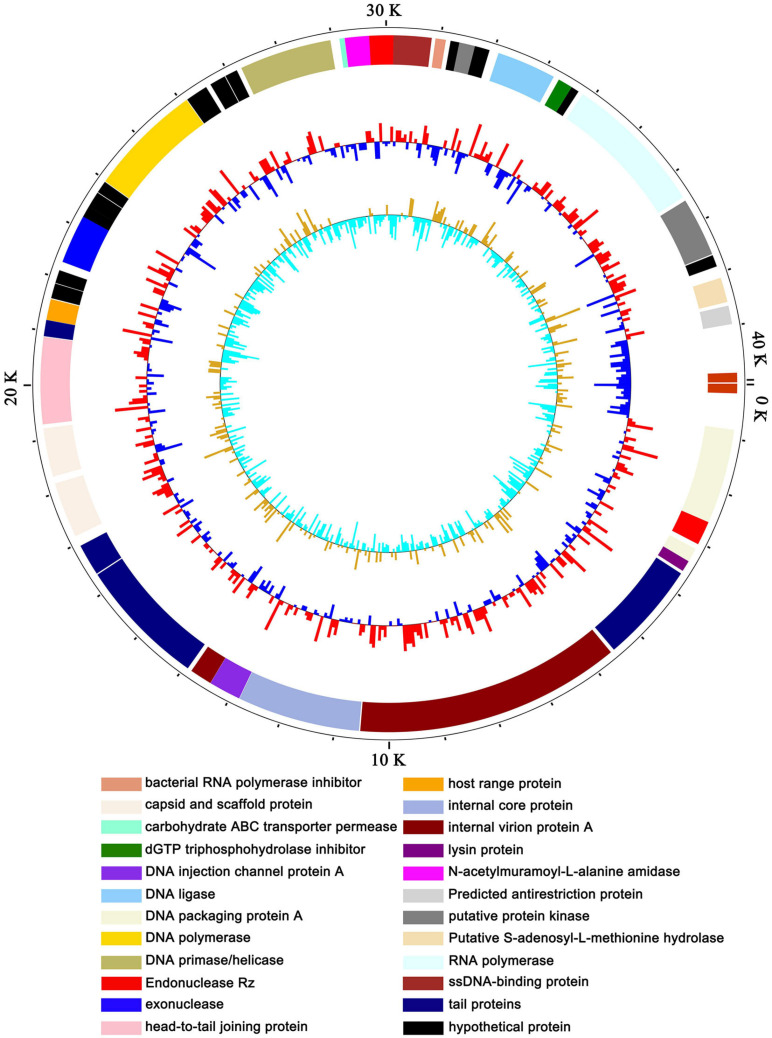
Map of the genome organization of bacteriophage vB_ShiP-A7. The predicted ORFs of vB_ShiP-A7 are indicated in different colors in the outermost ring. All of the ORFs predicted in the vB_ShiP-A7 genome were in negative strand. ORFs with predicted functions are marked in different colors. Hypothetical proteins are labelled in black. The second ring represents the GC content. Red outward indicates that the GC content is above average, and blue inward indicates that the GC content is below average. The innermost ring represents the GC skew.

To exclude the possibility that vB_ShiP-A7 contains any virulent proteins, it is necessary to identify the complete genome sequence of vB_ShiP-A7. Next-generation sequencing results showed that the complete genome of phage vB_ShiP-A7 is a linear double-stranded DNA of approximately 39,881 bp. To verify the nucleotide sequence obtained, the genomic DNA of phage vB_ShiP-A7 was further digested with EcoRI (2 sites at 28,588 and 37,095), PstI (1 site at 9333) (Supplementary Figure 1). However, 2 unexpected 2.9 kb and 9.3 kb fragments were identified after digestion, indicating that phage vB_ShiP-A7 is likely linear but not circular. To determine the terminal sequences of the vB_ShiP-A7 genome, the 2.9 kb and 9.3 kb fragments obtained by EcoRI and PstI digestion were recovered and directly sequenced with outward-pointing primers. As expected, the sequencing reaction terminated at the end of the genome. Thus, the vB_ShiP-A7 genome is indeed linear and has identical direct terminal repeats (DTRs) of 177 bp. We proved that the genomic DNA of vB_ShiP-A7 contains 177 bp terminal repeats located in the genome from nucleotides 1–177 and 39,882–40,058 (Figure 3). Therefore, the final genome length of phage vB_ShiP-A7 is 40,058 bp with 49.4% GC content (Table 2 and Figure 3). The general organization of the genome of vB_ShiP-A7 follows that of the T7-like phages, in which 43 putative ORFs were predicted in the complementary strand, as summarized in Table 2. We did not find tRNA or rRNA genes in the genome of vB_ShiP-A7. Thirty-one ORFs (72.1%) were highly homologous to known functional genes, which were predicted to have similar functions with related genes (Table 2) and are labeled in different colors in Figure 3. The predicted functional proteins encoded by vB_ShiP-A7 can be divided into five categories: DNA/RNA replication/modification (DNA polymerase, DNA primase/helicase, ssDNA-binding protein, DNA ligase, RNA polymerase, bacterial RNA polymerase inhibitor, nucleotide kinase, exonuclease, and endonuclease), host lysis (lysin protein and endopeptidase Rz), packaging (DNA packaging protein and DNA packaging protein A), structural proteins (tail fiber protein, internal virion protein D, internal core protein, DNA injection channel protein A, internal virion protein A, tail tubular protein A, tail tubular protein B, major capsid protein, capsid and scaffold protein, head-to-tail joining protein, tail assembly protein, and host range protein), and additional functions (carbohydrate ABC transporter permease, N-acetylmuramoyl-L-alanine amidase, dGTP triphosphohydrolase inhibitor, putative protein kinase, putative S-adenosyl-L-methionine hydrolase, and predicted antirestriction protein) (Table 2, Figure 3). Twelve hypothetical proteins were also predicted in the genome of vB_ShiP-A7, and their potential functions in the life cycle of vB_ShiP-A7 need to be determined in future studies. In addition, lysogen-related proteins, such as integrase, recombinase, repressor, and excisionase, were not present in the genome of vB_ShiP-A7. We conclude that phage vB_ShiP-A7 is a lytic bacteriophage. We did not observe any known toxic proteins encoded by the genome of vB_ShiP-A7. The characteristics of this lytic phage lacking genes encoding harmful factors make it an effective and promising candidate as an antibacterial agent.

**TABLE 2:** Predicted ORFs and genes encoded by the vB_ShiP-A7 genome (GenBank database under accession number MK685668).

**ORFs**	**Start**	**Stop**	**Directions**	**No of residues**	**MW(da)**	**pI**	**Predicted molecular function**
ORF1	848	2608	-	586	66236.1	5.35	DNA packaging protein
ORF2	2608	3054	-	148	16740.9	7.51	endopeptidase Rz
ORF3	3153	3419	-	88	10113.7	4.38	DNA packaging protein A
ORF4	3412	3618	-	68	7337.2	6.72	lysin protein
ORF5	3675	5540	-	621	68063.2	6.34	tail fiber protein
ORF6	5612	10546	-	1644	179456.2	7.02	internal virion protein D
ORF7	10570	12849	-	759	85637.0	5.78	internal core protein
ORF8	12856	13449	-	197	21320.4	10.26	DNA injection channel protein A
ORF9	13452	13865	-	137	15747.5	8.71	internal virion protein A
ORF10	13946	16342	-	798	89101.9	6.35	tail tubular protein B
ORF11	16365	16955	-	196	21966.7	4.22	tail tubular protein A
ORF12	17154	18188	-	344	36520.6	6.63	major capsid protein
ORF13	18321	19220	-	299	32722.7	3.91	capsid and scaffold protein
ORF14	19295	20902	-	535	58734.0	4.35	head-to-tail joining protein
ORF15	20916	21218	-	100	10201.8	10.64	tail assembly protein
ORF16	21221	21607	-	128	14558.0	6.78	host range protein
ORF17	21626	21904	-	92	9895.3	10.26	hypothetical protein
ORF18	21914	22159	-	81	9134.7	4.40	hypothetical protein
ORF19	22322	23236	-	304	34816.3	4.79	exonuclease
ORF20	23233	23442	-	69	7319.0	9.87	hypothetical protein
ORF21	23442	23744	-	100	11218.9	8.50	hypothetical protein
ORF22	23756	23983	-	75	9410.1	6.24	hypothetical protein
ORF23	23997	26111	-	704	79084.7	6.33	DNA polymerase
ORF24	26124	26540	-	138	15743.3	9.66	hypothetical protein
ORF25	26632	26934	-	100	10943.6	10.65	hypothetical protein
ORF26	26948	27181	-	77	8715.8	11.71	hypothetical protein
ORF27	27273	28985	-	570	63571.7	4.89	DNA primase/helicase
ORF28	29153	29245	-	30	3665.0	7.53	carbohydrate ABC transporter permease
ORF29	29250	29705	-	151	16950.3	8.10	N-acetylmuramoyl-L-alanine amidase
ORF30	29702	30148	-	148	17215.9	9.80	endonuclease
ORF31	30148	30852	-	234	26229.4	4.24	ssDNA-binding protein
ORF32	30922	31113	-	63	6972.6	4.93	bacterial RNA polymerase inhibitor
ORF33	31201	31374	-	57	6888.7	4.55	hypothetical protein
ORF34	31364	31657	-	97	11374.1	5.21	nucleotide kinase
ORF35	31657	31935	-	92	10188.1	11.30	hypothetical protein
ORF36	32108	33208	-	366	41577.2	4.68	DNA ligase
ORF37	33316	33588	-	90	10648.3	7.51	dGTP triphosphohydrolase inhibitor
ORF38	33588	33737	-	49	6078.8	11.37	hypothetical protein
ORF39	33829	36480	-	883	98990.8	7.32	RNA polymerase
ORF40	36554	37651	-	365	41750.0	7.61	putative protein kinase
ORF41	37673	37891	-	72	8446.1	11.76	hypothetical protein
ORF42	38113	38571	-	152	17040.3	6.74	putative S-adenosyl-L-methionine hydrolase
ORF43	38640	38984	-	114	13287.4	3.71	predicted antirestriction protein

### Comparative Genome Analysis of vB_ShiP-A7 With Its Related Phages

**FIGURE 4:**
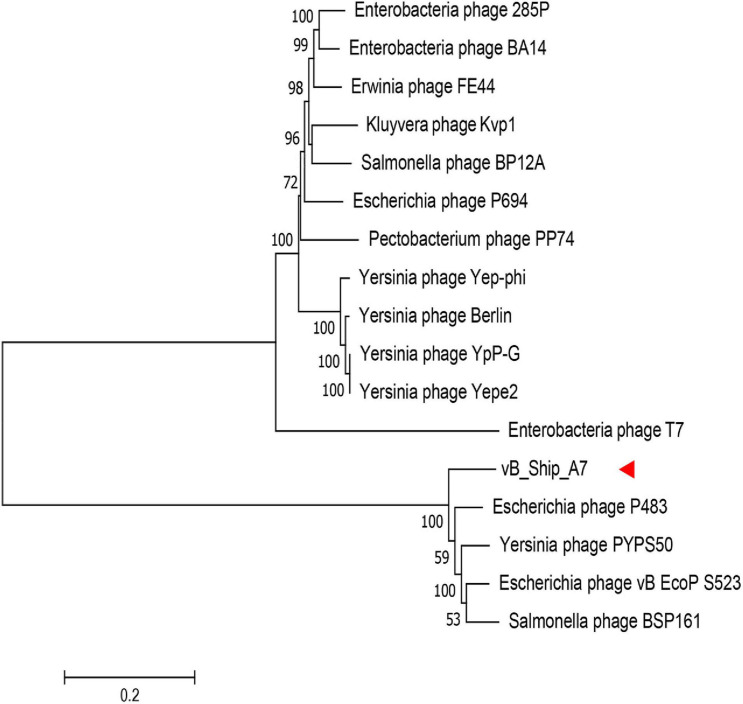
Phylogenetic tree based on the whole genome sequence of vB_ShiP-A7 and its related phages. After comparing the genome sequences of these phages using the ClustalW program, a phylogenetic tree was generated using the neighbour-joining method with 1000 bootstrap replicates.vB_ShiP-A7 was marked by a red arrow.

The whole-genome sequence of vB_ShiP-A7 was analyzed using BlastN analysis against the NCBI nonredundant DNA database. The highest similarity throughout the whole vB_ShiP-A7 genome was observed with 15 phages (coverage 74%–88%, identity 86%–93%), including *Escherichia* phage P483 (Chen et al., 2016), *Escherichia* phage P694 (Chen et al., 2016), *Enterobacteria* phage BA14 (Mertens and Hausmann, 1982), *Escherichia* phage vB EcoP S523, *Enterobacteria* phage 285P,*Pectobacterium* phage PP74 (Kabanova et al., 2018), *Kluyvera* phage Kvp1 (Lingohr et al., 2008), *Erwinia* phage FE44 (Faidiuk and Tovkach, 2014), *Salmonella* phage BSP161, *Salmonella* phage BP12A, *Yersinia* phage PYPS50, *Yersinia* phage Yepe2,*Yersinia* phage YpP-G (Rashid et al., 2012), *Yersinia* phage Berlin and *Yersinia* phage Yep-phi (Zhao et al., 2011) (Figure 4). Except for *Escherichia* phage vB EcoP S523, *Salmonella* phage BSP161, *Salmonella* phage BP12A, and *Yersinia* phage PYPS50, all the other 11 related phages are known T7-like phages. To further explore the evolutionary position of vB_ShiP-A7, a phylogenetic analysis was performed with MEGA using the neighbor-joining method for the genomes of phage T7, phage vB_ShiP-A7, and its related 15 different phages. The phylogenetic tree based on the whole genomes of these phages shows 2 major branches (Figure 4). vB_ShiP-A7 is closely related to phages *Escherichia* phage P483, *Escherichia* phage vB EcoP S523, *Yersinia* phage PYPS50, and *Salmonella* phage BSP161 (Figure 4). We also built a phylogenetic tree based on one of the conserved proteins (major capsid protein) of these phages. The result suggested that phage vB_ShiP-A7 is most closely related to *Pectobacterium* phage PP74 and *Escherichia* phage P694 and is relatively close to phage T7 (Supplementary Figure 2). In addition, the genome organization of vB_ShiP-A7 follows that of the T7-like phages. The genomic information indicates that phage vB_ShiP-A7 is a novel member of the unclassified T7-like phages. Currently, this group of phages is highly variable. vB_ShiP-A7 and its related phages are isolated from different places in the world, and they can infect different species of bacteria, suggesting that the evolutionary relationships among these phages are complicated.

The DTRs of vB_ShiP-A7 are very similar to the DTRs of Berlin, Yepe2, Yep-phi, and Kvp1 (Lingohr et al., 2008; Zhao et al., 2011; Chen et al., 2016). The diversity of packaging mechanisms of phages, leading to various types of DNA termini, i.e., 5′cos (Lambda), 3′cos (HK97), pac (P1), headful without a pac site (T4), DTR (T7), and host fragment (Mu), has been described (Garneau et al., 2017). The vB_ShiP-A7 genome has DTRs of 177 bp, and similar DTRs were also found in its genetic relatives and the T7 phage, indicating that phage vB_ShiP-A7 uses a similar packaging mechanism as in the T7 phage.

**FIGURE 5:**
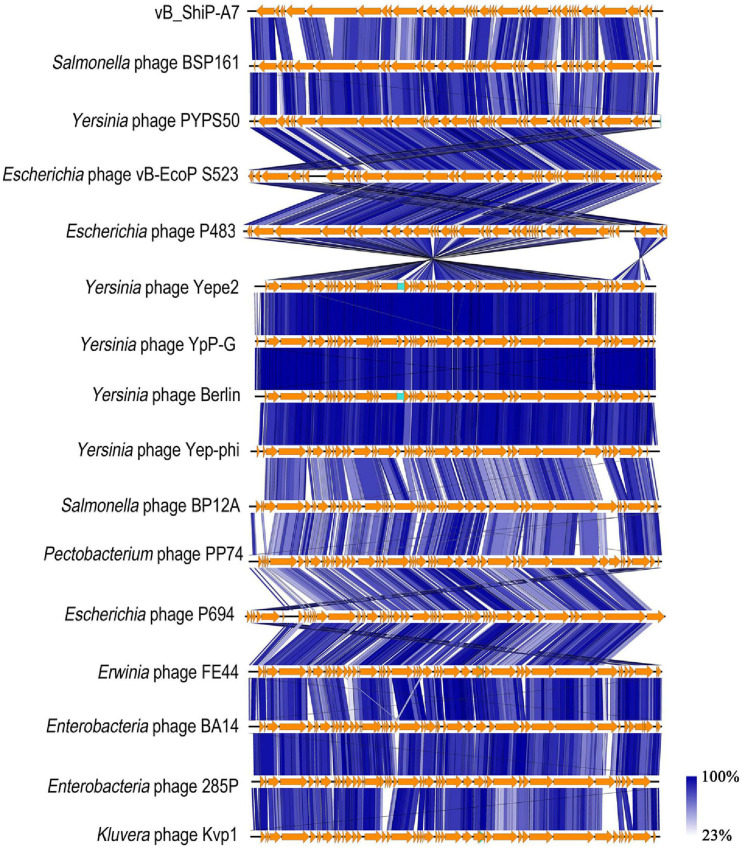
Schematic genomic alignment of phage vB_ShiP-A7 with its related phages. Homologous ORFs or genes are present in brown, and amino acid identities of different proteins are indicated by different shades of blue.

We compared proteins encoded by phage vB_ShiP-A7 with that of its 15 related phages using EasyFig. Most of the proteins encoded by vB_ShiP-A7 and its 15 related phages are highly similar (Figure 5). Several dissimilar proteins among the genomes of these phages are shown as colorless or in light colors in Figure 5. DNA ligase encoded by ORF36 of vB_ShiP-A7 shows divergence with related phages (100% coverage, 65%–74% identity). The tail assembly protein encoded by ORF15 of vB_ShiP-A7 is very different from the homologous proteins of the other related phages (45%–96% coverage and 57%–89% identity). The tail fiber protein encoded by ORF5 of vB_ShiP-A7 also has a relatively low homology with that of its related phages (37%–46% coverage and 54%–60% identity) (Figure 5). The similarity of the tail fiber protein of vB_ShiP-A7 with other homologs is only found at the N-terminus, which is associated with the tail structure (Lingohr et al., 2008). The C-terminus of this tail fiber protein of vB_ShiP-A7, involved in ligand interactions, exhibits relatively large variability from tail fiber proteins of related phages (Figure 5). We built a phylogenetic tree based on the tail fiber proteins of different phages (Supplementary Figure 3). The tail fiber protein of this phage is most similar to that of *Yersinia* phage VB_YenP-AP5 and *Serratia* phage 2050H2, and it is relatively close to the tail fiber protein of *Escherichia* phage P694. The variation in tail fiber protein is relatively large, and it is very different from the major capsid protein in evolution.

### Structural Proteins of vB_ShiP-A7

**FIGURE 6:**
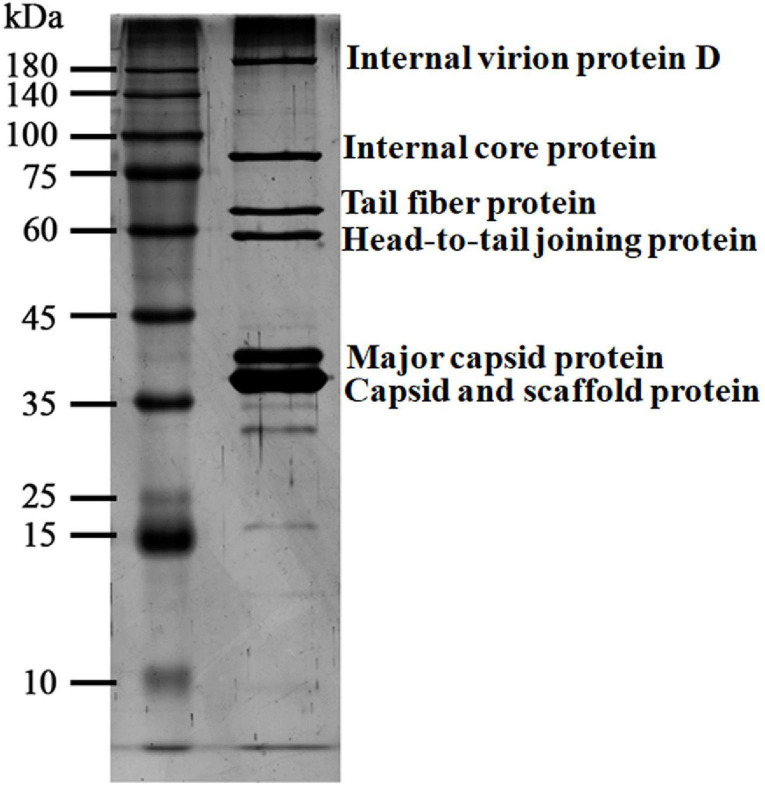
Particle proteins of vB_ShiP-A7 separated by SDS-PAGE gel. The purified vB_ShiP-A7 particles were separated by SDS-PAGE and stained with silver. The six bands from top to bottom corresponding to different particle proteins of vB_ShiP-A7 are internal virion protein D, internal core protein, tail fiber protein, head-to-tail joining protein, major capsid protein, capsid, and scaffold protein. Protein molecular mass markers are indicated on the far left.

Purified phage vB_ShiP-A7 particles were denatured and separated using SDS-PAGE. At least six distinct protein bands were shown in the silver-stained SDS-PAGE gel, which were speculated to be structural proteins of vB_ShiP-A7 by their estimated molecular weights (internal virion protein D, internal core protein, tail fiber protein, head-to-tail joining protein, major capsid protein, capsid, and scaffold protein) (Figure 6). To further confirm these structural proteins, the proteins of the phage particles were analyzed using mass spectrometry (Table 3). A total of 18 proteins were identified, including all the proteins shown on the SDS-PAGE gels (Table 3, Figure 6). Nine of them are known structural proteins, and DNA primase/helicase was also identified in the phage particles. In addition, some hypothetical proteins (ORF17, ORF18, A7_225, A7_120, A7_146, A7_426, A7_68, and A7_88) were detected using mass spectrometry, but their functions need to be determined further. The hypothetical proteins encoded by A7_225, A7_120, A7_146, A7_426, A7_68, and A7_88 were only found when we used all of the possible ORFs predicted with Artemis in the genome of vB_ShiP-A7 as a reference. However, they were omitted from the annotation file of vB_ShiP-A7 (uploaded to NCBI under assigned number MK685668) since they do not have similar sequences to any predicted proteins at NCBI (see text footnote 1) or these ORFs may exist in the interior of known genes. Interestingly, some of these peptides (A7_225, A7_120, A7_146, A7_68, and A7_88) were encoded by antisense RNAs on known genes of the late operon of vB_ShiP-A7 (Table 3). In addition, proteins that were similar to the known toxic proteins through a comparative analysis were not identified *via* mass spectrometry in vB_ShiP-A7 particles.

**TABLE 3:** Phage vB_ShiP-A7 particle proteins identified in mass spectrometry.

**First protein**	**Unique peptides**	**Unique sequence coverage [%]**	**Mol. weight [kDa]**	**Sequence length**	**Start**	**End**	**Location**
ORF6	111	72.9	179.52	1644	10546	10546	ORF6
ORF7	56	69.7	85.783	760	12852	12852	ORF7
ORF12	34	92.6	37.502	351	18188	18188	ORF12
ORF14	25	56.6	58.744	535	20902	20902	ORF14
ORF13	15	42.5	33.488	306	19220	19220	ORF13
ORF8	11	65.2	22.545	207	13449	13449	ORF8
ORF15	7	66	10.875	106	21218	21218	ORF15
ORF17	4	51.1	9.8971	92	21904	21904	ORF17
A7_225	3	25.4	7.7616	67	29770	29970	Antisense of ORF30
A7_120	2	28.1	10.122	89	16521	16787	Antisense of ORF11
A7_146	2	39	6.5657	59	19923	20099	Antisense of ORF14
ORF27	2	5.3	64.9	582	28985	27273	ORF27
ORF18	2	43.9	9.2674	82	22159	21914	ORF18
A7_426	2	46.7	3.5044	30	19069	18980	Antisense of ORF13
ORF11	2	18.4	21.97	196	16955	16365	ORF11
ORF5	2	4.7	68.075	621	5540	3675	ORF5
	2	93	4.7304	43	9639	9767	Antisense of ORF6
A7_88	2	34.8	7.6992	69	11963	12169	Antisense of ORF7

### The Ability of vB_ShiP-A7 to Destroy Bacterial Biofilms

**FIGURE 7:**
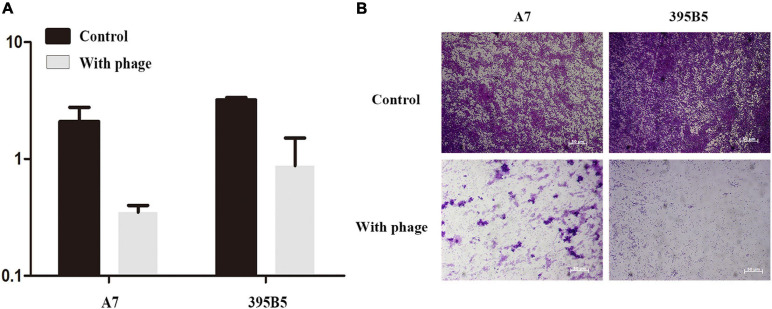
Crystal violet assay showing the biofilm biomass of *Shiglla flexneri* A7 and *E. coli*395B5 grown in the presence or absence of phage vB_ShiP-A7. **(A)** We measured optical density readings in replicates to evaluate the inhibition of biofilm by phage vB_ShiP-A7. **(B)** The 2 above pictures are *Shiglla flexneri* A7 strain and *E. coli* 395B5 strain without phage; The bottom 2 pictures are *Shiglla flexneri* A7 and *E. coli* 395B5 exposured to phage vB_ShiP-A7.

Biofilm removal is the key to treating chronic infectious diseases. We tested the effects of phage vB_ShiP-A7 on biofilms formed by *S. flexneri* A7 and *E. coli* 395B5. Approximately 24 h after the addition of 100 μL of phage vB_ShiP-A7 (10^9^ PFU/mL), the log reduction in biofilm biomasses of strains *S. flexneri* A7 and *E. coli* 395B5 due to phage treatment is shown in Figure 7A, indicating that this phage could inhibit biofilm formation. The biofilm formation on the surface of the plate wells of strains *S. flexneri* A7 and *E. coli* 395B5 in the phage-treated samples was significantly lesser than that of the untreated controls (Figure 7B). This result suggests that vB_ShiP-A7 can reduce biofilm formation on clinical strains of *S. flexneri* A7 and *E. coli* 395B5, indicating the possibility of using phage vB_ShiP-A7 as a biofilm disruption agent.

### vB_ShiP-A7 Administration Significantly Reduces *S. flexneri* A7 Colonization

**FIGURE 8:**
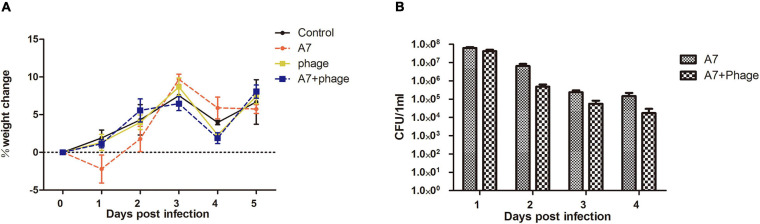
Inhibitory effect of phage vB_ShiP-A7 on *Shiglla flexneri* A7 infection in mice. **(A)** Changes of body weight of mice in different treatment groups, Control (control group), A7 (*Shiglla flexneri* A7infection group), phage (phagevB_ShiP-A7 group), and A7+phage (*Shiglla flexneri* A7 infection plus phage vB_ShiP-A7 treatment group). **(B)** Killing effect of the phage on *Shiglla flexneri* A7 in mice at different days after infection, A7 (*Shiglla flexneri* A7infection group) and A7+phage (*Shiglla flexneri* A7 infection plus phage vB_ShiP-A7 treatment group).

The mouse infection model results showed that the mice developed diarrhea symptoms on the first day after infection with *Shigella*. Three days after infection, the weight of the mice in the “*Shigella* infection group” was lower than that of the “control group,” which was consistent with the time of diarrhea symptoms. Phage vB_ShiP-A7 (“phage group”) had no effect on the health of the mice (Figure 8A). Compared with the “control group,” the weight of the mice in the “*Shigella* infection plus phage treatment group” had no significant change, indicating that phage vB_ShiP-A7 had a protective effect on *Shigella* infection in mice (Figure 8A).Next, 10^11^ purified vB_ShiP-A7 particles were added to drinking water to treat *Shigella* infection in mice. By counting the number of bacteria, we observed that the number of *Shigella* cells in the feces of the “*Shigella* infection plus phage treatment group mice” was significantly (3–10 folds) reduced compared to that in the “*Shigella* infection group”(Figure 8B), suggesting that the phage had a certain bactericidal effect on mice *in vivo*.

## Discussion

*Shigella* spp. and *E. coli* belong to the family *Enterobacteriaceae*, which can cause intestinal infections (The et al., 2016). Previous studies have demonstrated that bacteriophages can be used to treat these infections (Soffer et al., 2017; Tomat et al., 2018). In this study, a new lytic phage, vB_ShiP-A7, was isolated and characterized, with lytic activity against several MDR clinical strains of both *S. flexneri* and *E. coli* (Table 1). Several phages have been reported to have the ability to infect both *E. coli* and *Shigella* (Chang and Kim, 2011; Lee et al., 2016; Sváb et al., 2019). Since *E. coli* and *Shigella* are genetically similar, it is reasonable to expect some similar phages to target both strains. However, not all phages can lyse *Shigella* and *E. coli* at the same time. Phage SSE1 against *S. dysenteriae* has the highest identity with *Escherichia* phage ST0, but it cannot infect *E. coli* ST100 strains (Lu et al., 2020). Lytic phages are relatively specific and usually infect a subgroup of strains within one bacterial species or across closely related species, which causes less disruption to the gut flora than antibiotic treatment (Zhang et al., 2013). Phage vB_ShiP-A7 can infect some specific strains of *S. flexneri* and *E. coli* (Table 1). The EOPs of this phage for all the test strains are shown in the last column of Table 1, indicating that the phage can infect and lyse some serotypes of *S. flexneri* clinical strains more efficiently than *E. coli* clinical strains. However, phage vB_ShiP-A7 does not recognize the wild-type *E. coli* strain MG1655.

Phage vB_ShiP-A7 belongs to the family *Podoviridae*, based on its morphology under an electron microscope (Figure 1). vB_ShiP-A7 has a large burst size, suggesting that the concentration of bacteriophage vB_ShiP-A7 increased rapidly and effectively lysed bacteria (Nilsson, 2014). Next-generation sequencing demonstrated that the genome of phage vB_ShiP-A7 does not encode integrases, recombinases, or harmful gene products (Table 2, Figure 3). In addition, phage vB_ShiP-A7 showed promising effects against bacterial growth in liquid and biofilm (Figures 2, 6), suggesting that it may be used as an anti-infective agent.

The general genome organization of phage vB_ShiP-A7 is similar to that of T7-like phages, which encode RNA polymerase. Comparative genomic analysis demonstrated that phage vB_ShiP-A7 is highly similar to 15 related phages, among which 11 phages are unclassified T7-like phages (Figures 4, 5). The three considerably different phylogenetic trees based on whole-genome sequences of phages, major capsid proteins, and tail fiber proteins suggest that phage vB_ShiP-A7 is closely related to T7-like phages (Figure 4, Supplementary Figures 2, 3). Therefore, this phage can be assigned as a novel virulent phage of the T7-like family. Several proteins of this phage are different from those of other phages, especially the tail fiber protein (Figure 5, Supplementary Figure 3). The tail protein of phages is the key protein that recognizes host bacteria. Different tail proteins of phages can cause phages to infect different host bacteria (Steven et al., 1988). Yosef et al. (2017) reported that a small change in the tail protein sequence of a phage can lead to changes in the host range. The different C-terminus of the tail fiber protein of vB_ShiP-A7, involved in ligand interactions, may allow this phage to infect some specific hosts. The phage could be used as a component of a phage cocktail. Most of the genes of vB_ShiP-A7 and the 15 related phages are highly homologous. High homology of the same functional phage genes was found in different phage species, indicating that horizontal gene transfer between phages is a component of evolution (Chen et al., 2016; Yosef et al., 2017). The gene arrangement of phage vB_ShiP-A7 is different from that of its related phages (Figure 5). Gene rearrangement was also observed in other *Escherichia* phages (Xu et al., 2018). vB_ShiP-A7 and its relatives may have evolved through horizontal exchange and rearrangement of their genes, which is a common phenomenon in the evolution of tailed phages (Zhao et al., 2011; Dekel-Bird et al., 2013; Chen et al., 2016).

Eighteen proteins were identified in vB_ShiP-A7 phage particles using mass spectrometry, including known structural proteins and hypothetical proteins (Table 3, Figure 6). Interestingly, some of these hypothetical proteins were encoded by antisense RNA on late operon-encoded structure proteins (A7_225, A7_120, A7_146, A7_68, and A7_88) (Table 3). Anne et al. found that PAK_P3 expressed antisense RNA elements targeting its structural region during the early stage of infection (Chevallereau et al., 2016), which might be used to shut down the expression of late structural genes during the early stage of infection. An antisense RNA was also found in the lambda phage genome, which was transcribed from the paQ promoter and did not encode proteins or peptides (Nejman-Faleńczyk et al., 2015). We found several small peptides/proteins encoded by antisense RNAs in *E. coli* phage vB_EcoP-EG1 (Gu et al., 2019). These small peptides/proteins encoded by antisense RNAs in the late operon may exist in different phages and are involved in phage infection processes, and their detailed functions remain to be determined.

In terms of controlling bacterial food source pollution, some bacteriophage products have been certified by FDA, such as ListShield^TM^, EcoShield^TM^, and SalmoFresh^TM^ (Tang et al., 2019). Currently, ShigaShield^TM^ against *Shigella* is undergoing FDA and USDA reviews for their safety (Soffer et al., 2017). There is no mature commercial phage for the treatment of *Shigella dysentery* (Tang et al., 2019). A large number of *Shigella* phages with clear properties are needed for the preparation of phage cocktails with clinical application value. Before a new phage is used in human infection treatment, its safety, antibacterial effect, phage pharmacology, and infection kinetics must be tested in animal models, which will improve our understanding of the pharmacology, immunology, safety, and potential for bacterial resistance (Romero-Calle et al., 2019). *Shigella* spp. infection in mice cannot completely simulate human dysentery (Mai et al., 2015), but it can still give us some useful information on evaluation of phage lysis efficiency against *Shigella* and the toxicity of phages *in vivo*. We found that *Shigella* only caused transient diarrhea in mice. Phage vB_ShiP-A7 treatment reduced the number of *Shigella* cells in the intestines of these mice, and the mice in the phage treatment group neither suffered transient diarrhea nor experienced weight loss. The results showed that phage vB_ShiP-A7 had a therapeutic effect on mice infected with MDR *Shigella*, and no adverse reactions were observed. The phage may be used to treat infections caused by drug-resistant *Shigella* spp. Animal infection models can provide reference data for phage therapy, but the frequency and duration of phage treatment, route of administration, and optimal dose need to be verified through preclinical trials (Wittebole et al., 2014). Thus, vB_ShiP-A7 can be used to treat human infectious diseases only after human preclinical trials.

## Conclusion

In this study, a novel phage, vB_ShiP-A7, was isolated and fully characterized. The phage was stable and could rapidly lyse its host bacteria. The phage genome lays a foundation for studying the interaction between phages and their hosts. Comparative genomic analysis of this phage with related phages sheds light on the mechanisms of evolutionary changes in these T7-like family phage genomes. *In vitro* and *in vivo* experiments showed that the phage could effectively control the number of drug-resistant bacteria. Given bacteriophages have been utilising gradually in the food processing industry, or in human disease treatment (Tang et al., 2019), this phage we isolated and characterized may become an alternative treatment for infections caused by MDR *Shigella* and *Escherichia*, hence reducing the pressure to find new antibiotics.

## Data Availability Statement

The datasets presented in this study can be found in online repositories. The names of the repository/repositories and accession number(s) can be found below: https://www.ncbi.nlm.nih.gov/genbank/, MK685668.

## Ethics Statement

The studies involving human participants were reviewed and approved by the ethical rules of Nanjing Medical University (Nanjing, China), The First Affiliated Hospital of Nanjing Medical University, and Jiangsu Provincial Center for Disease Control and Prevention (Nanjing, China), and informed consent was obtained from all patients. The patients/participants provided their written informed consent to participate in this study. The animal study was reviewed and approved by the ethical rules of Nanjing Medical University. Written informed consent was obtained from the individual(s) for the publication of any potentially identifiable images or data included in this article.

## Author Contributions

JX, RZ, XY, and XL conceived, designed, and coordinated the study. JX, RZ, and XY carried out the experimentation. JX and XL analyzed the data. GL, JX, XY, and XL contributed to the reagents, materials, and analysis tools. XZ helped to edit the manuscript. All authors read and approved the manuscript.

## Conflict of Interest

The authors declare that the research was conducted in the absence of any commercial or financial relationships that could be construed as a potential conflict of interest.

## Publisher’s Note

All claims expressed in this article are solely those of the authors and do not necessarily represent those of their affiliated organizations, or those of the publisher, the editors and the reviewers. Any product that may be evaluated in this article, or claim that may be made by its manufacturer, is not guaranteed or endorsed by the publisher.

## Footnotes

1
http://blast.ncbi.nlm.nih.gov/Blast.cgi

2
http://lowelab.ucsc.edu/tRNAscan-SE

3
http://www.cbs.dtu.dk/services/RNAmmer/

4
http://mjsull.github.io/Easyfig/files.html

## Supplementary Material

Supplementary Figure 1 The terminal sequence of Phage vB_ShiP-A7 were determined by genomic digestion and sequencing.

Supplementary Figure 2 Phylogenetic tree based on the major capsid proteins’ sequence of vB_ShiP-A7 and its related phages.

Supplementary Figure 3 Phylogenetic tree based on the tail fiber proteins’ sequence of vB_ShiP-A7 and its related phages.

Supplementary Table 1 We determined the adsorption rate of this phage.
