# Supplementary material for: Molecular Characteristics of Novel Phage vB_ShiP-A7 Infecting Multidrug-Resistant Shigella flexneri and Escherichia coli, and Its Bactericidal Effect in vitro and in vivo
Source: Front Microbiol. 2021 Aug 26;12:698962. doi: 10.3389/fmicb.2021.698962 (PMC8427288; doi:10.3389/fmicb.2021.698962)
Supplement: Supplementary Figure 1 — The terminal sequence of Phage vB_ShiP-A7 were determined by genomic digestion and sequencing. [file Data_Sheet_1.docx]

**Supplementary Table1**

| Bacteriophage | Initial phage concentration (Incubation time, 0 min) | Free phage concentration  (Incubation time, 5 min) | Adsorption constant, k | % of adsorbed phages |
| --- | --- | --- | --- | --- |
| vB_ShiP-A7 | 996000/ml | 80000/ml | 1.405×10^-8^ | 91.97% |

**Supplementary Figures**

**Supplementary Figure 1.** The terminal sequence of Phage vB_ShiP-A7 were detemined by genomic digestion and sequencing. A. There are two EcoRI cut sites in the vB_ShiP-A7’s genome, but the genome is cut by EcoRI enzyme to form three different DNA fragments, indicated the phage genome is linear, and the smallest fragment located at one end of the genome was purified and sequenced to confirm one terminal sequence of the phage genome. 1kb DNA ladder on the left line. B. There are one PstI site in the vB_ShiP-A7’s genome, but the genome is cut by PstI enzyme to form two different DNA fragments. It confirmed the genome of vB_ShiP-A7 is linear, and the smaller fragment located at the other end of vB_ShiP-A7’s genome was purified and sequenced to confirm the other terminal of the genome.


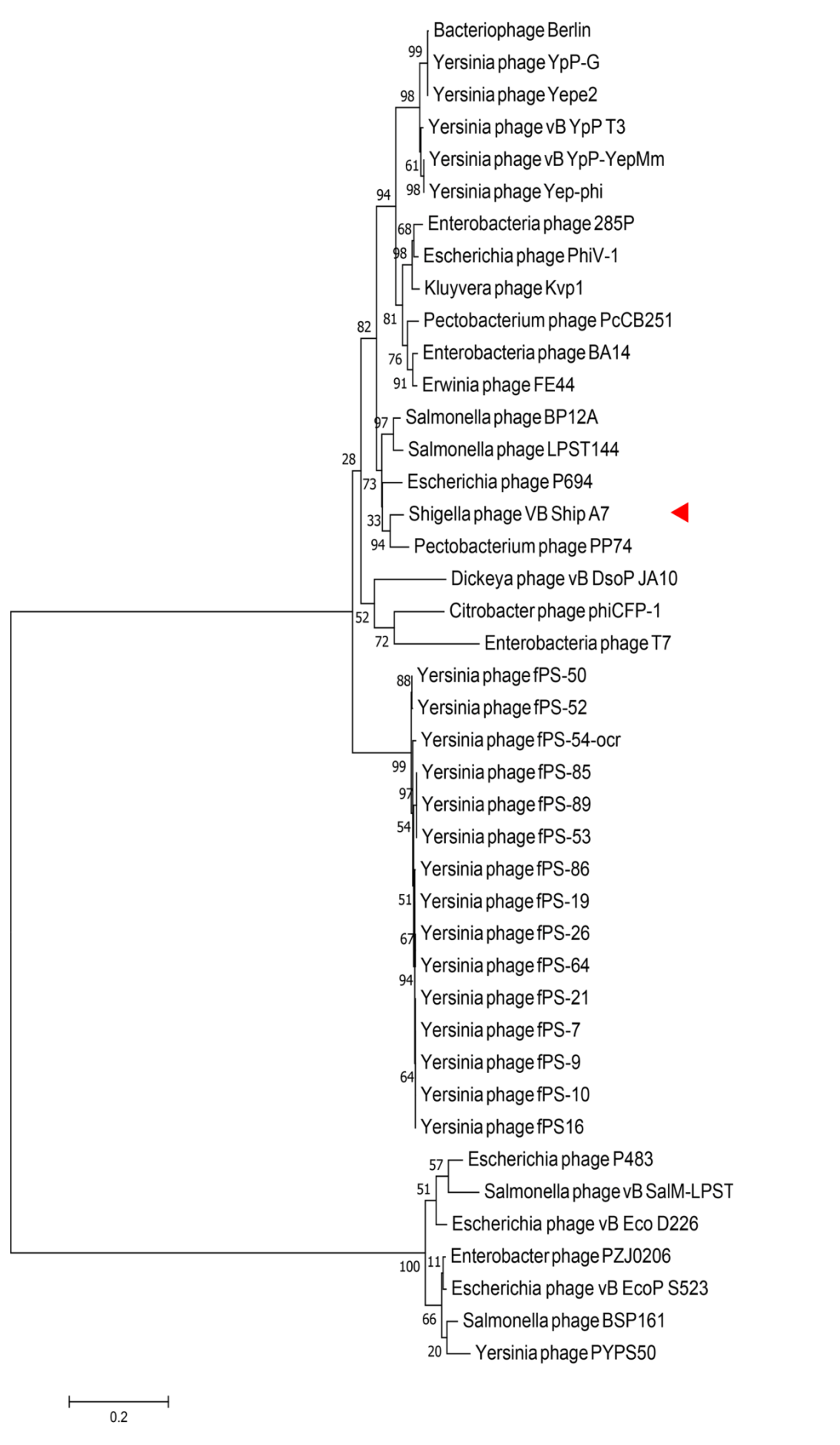


**Supplementary Figure 2.** Phylogenetic tree based on the major caspid proteins’ sequence of vB_ShiP-A7 and its related phages. After comparing the genome sequences of major caspid proteins using the ClustalW program, a phylogenetic tree was generated using the neighbour-joining method with 1000 bootstrap replicates. vB_ShiP-A7 was marked by a red arrow.


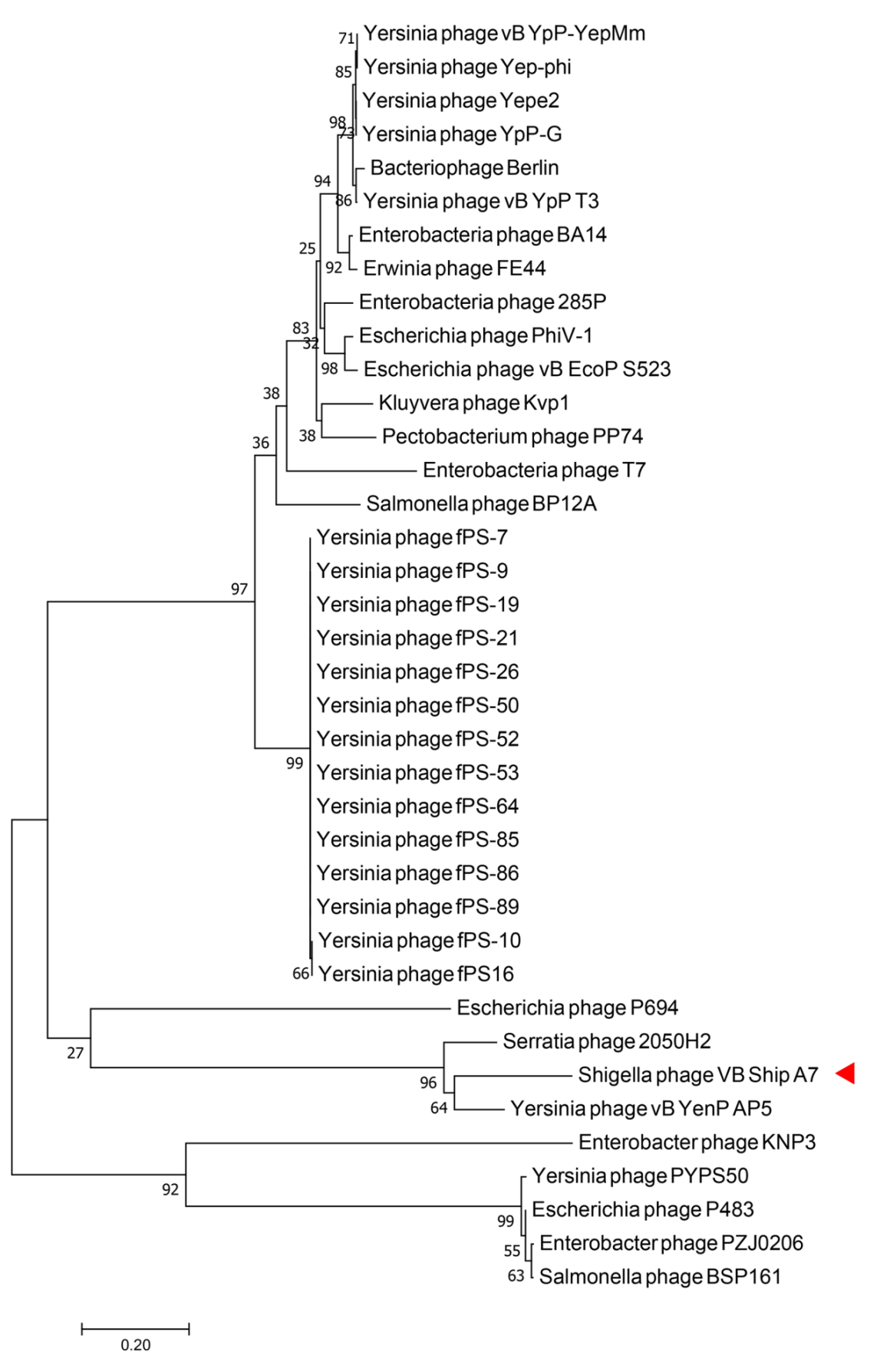


**Supplementary Figure 3.** Phylogenetic tree based on the tail fiber proteins’ sequence of vB_ShiP-A7 and its related phages. After comparing the genome sequences of tail fiber protein using the ClustalW program, a phylogenetic tree was generated using the neighbour-joining method with 1000 bootstrap replicates. vB_ShiP-A7 was marked by a red arrow.
